# Optimization of concentration-directed blood purification therapy in the management of diquat poisoning

**DOI:** 10.3389/fmed.2025.1713595

**Published:** 2025-11-19

**Authors:** Weiwei Qian, Xuxin Xie, Bofu Liu, Chengtong He, Yu Cao

**Affiliations:** 1 Department of Emergency, Shangjinnanfu Hospital, West China Hospital, Sichuan University, Chengdu, Sichuan, China; 2 Department of Emergency, West China Hospital, Sichuan University, Chengdu, Sichuan, China

**Keywords:** diquat, acute diquat poisoning, optimization of concentration-directed, blood purification therapy, case series

## Abstract

Diquat, a non-selective herbicide extensively used in China, is characterized by moderate toxicity and the absence of a specific antidote. Acute diquat poisoning frequently leads to multiple organ dysfunction syndrome (MODS), posing significant clinical challenges. This study investigated the application and optimization of concentration-directed blood purification therapy in patients suffering from diquat poisoning complicated by MODS. We analyzed the clinical outcomes of four patients who underwent hemodialysis, hemoperfusion (HP), and other blood purification modalities. The results demonstrated that individualized treatment regimens—guided by real-time monitoring of plasma diquat concentrations—markedly enhanced therapeutic efficacy and improved patient prognoses. By dynamically tailoring blood purification protocols to the patients’ toxin levels, this study introduced a novel clinical intervention strategy for the management of acute diquat poisoning. These findings underscore the clinical value of concentration-guided blood purification therapy and offer important insights for its broader implementation in practice.

## 1 Introduction

Diquat, a non-selective defoliating herbicide with pyridine compounds as its active ingredients, has raised significant public health concerns due to its high toxicity in cases of acute exposure, especially in agricultural regions where the herbicide is extensively applied (1). The rapid onset of severe symptoms and the high mortality rate associated with diquat ingestion pose a serious threat to human health. Diquat poisoning, typically resulting from ingestion, induces local irritation and corrosive effects mainly through oxidative stress and inflammatory cascades, with symptoms including burning pain in the mouth, throat, chest, and abdomen, along with nausea, vomiting, and diarrhea. It is widely distributed in the body after absorption and is difficult to eliminate, leading to a high mortality rate (2). However, the effective treatment strategies for acute diquat poisoning remain limited due to the lack of a specific antidote for diquat.

Current treatments primarily revolve around decontamination, supportive care, and measures to curtail diquat absorption, further prompt and effective measures to prevent absorption or remove the toxin from the body are crucial for improving the prognosis of acute diquat poisoning (3). Given the complexity of acute diquat poisoning, involving multiple organ systems and complex pathophysiological processes, there is an urgent need for a multidisciplinary collaborative approach. This approach combines the expertise of various medical fields such as emergency medicine, toxicology, intensive care, nephrology, etc., Here, this case series aims to share our experience in managing 4 patients with acute diquat poisoning through multidisciplinary collaborative treatment, highlighting the importance of early intervention and comprehensive management (Table 1).

**TABLE 1:** Changer of laboratory results before and after HP/CRRT in four cases with acute diquat poisoning.

	Case 1						Case 2				Case 3					Case 4				
**Date**	**8.8**	**8.9**	**8.11**	**8.14**	**8.17**	**8.18**	**2.15**	**2.17**	**2.19**	**2.21**	**11.30**	**12.1**	**12.4**	**12.7**	**12.9**	**3.24**	**3.25**	**3.26**	**3.27**	**3.28**
**No.**	**Based**	**First**	**Second**	**Third**	**Fourth**	**Fifth**	**Based**	**First**	**Second**	**Third**	**Based**	**First**	**Second**	**Third**	**Fourth**	**Based**	**First**	**Second**	**Third**	**Fourth**
Day after admission	0	1	3	6	9	10	0	2	4	6	0	28	31	34	36	0	1	2	3	4
Exposure time (hours)	24	–	–	–	–	–	48	–	–	–	10	–	–	–	–	14	–	–	–	–
WBC count (10^9^/L)	27.72	7.55	6.12	16.98	–	35.5	17.76	11.59	8.97	–	18.26	8.32	17.48	17.85	23.66	18.43	18.91	–	10.12	–
NEU%	91.5	81	82.7	75	–	76	91.6	75.4	71	–	91.5	91.7	96.1	80	74	90.2	80	–	77.9	–
Hemoglobin (g/L)	169	141	122	85	–	74	126	97	78	–	72	64	73	79	68	155	144	–	118	–
Platelet (10^9^/L)	164	72	43	76	–	110	253	76	59	–	328	156	47	58	58	192	135	–	42	–
ALT (IU/L)	92	155	74	36	124	104	112	221	109	82	150	115	57	33	37	25	200	–	86	–
AST (IU/L)	174	94	19	17	62	36	216	63	30	39	114	83	22	25	35	24	233	–	52	–
TBIL (μmol/L)	15.1	18.8	7.5	5.7	11.8	9.5	10.5	4.3	4.2	2.9	6.3	5.4	11.3	17.3	9.6	22.7	16.7	–	5.5	–
GGT (IU/L)	43	26	–	63	212	190	21	28	21	24	16	14	19	89	156	21	14	–	25	–
Creatinine (μmol/L)	323	440	460	207	306	291	214	165	165	316	654	363	122	115	313	104	201	–	252	–
Urea (mmol/L)	9.5	15.7	16	10.4	20.8	22.7	9.7	3.9	3.6	12.1	15.6	9.6	2.4	3.7	16.4	3.4	4.1	–	3	–
CK-MB (ng/mL)	1.96	4.53	2.41	1.18	–	–	0.69	–	–	–	–	29.35	8.74	6.7	12.85	2.32	–	–	3.79	–
CTNT (ng/mL)	21.4	36.5	21	12.8	–	–	7	–	–	–	–	11.3	7.9	12.9	51.9	4	–	–	4	–
MYO (ng/mL)	224.3	254.5	239	158.3	–	–	114.2	–	–	–	–	>3000.00	1287	1755	>3000	64.51	–	–	334.9	–
NT-PRO-BNP (pg/mL)	590	1061	1782	1376	–	–	–	–	–	–	–	2650	520	341	3350	23	–	–	–	–
PT(s)	14.3	11.4	10.2	11.5	–	12.3	9.2	–	9.8	–	10.4	10.3	11	10.9	–	11	–	–	–	–
APTT(s)	123.5	74.9	39	32.9	–	33.5	22.2	–	26.5	–	28	29.6	37	32.3	–	37	–	–	–	–
DQ (ng/mL)	3125.47	951.65	94.94	22.53	–	–	5591.6	98.51	–	–	2475.1	759.7	165.2	63.1	89.3	4512.26	1126.09	137.58	90.46	38.81
Length of stay in ICU (days)	15						5				7					5				
Hospitalization (days)	34						10				10					10				
Prognosis	Survive						Survive				Death					Survive				

ALT, alanine aminotransferase; AST, aspartate aminotransferase; TBIL, total bilirubin; GGT, gamma-glutamyl transferase; WBC, white blood cell; NEU, neutrophil; PT, prothrombin time; APTT, activated partial thromboplastin time; CK-MB, creatine kinase–myocardial band; cTnT, cardiac troponin T; DQ, plasma diquat concentration; MYO, myoglobin; NT-proBNP, N-terminal pro–B-type natriuretic peptide; DQ, diquat; ICU, intensive care unit.

## 2 Methods

### 2.1 Literature review methodology

A structured literature review was conducted to gather relevant data on the clinical features, pathophysiology, and management of acute diquat poisoning. The databases searched included PubMed, Embase, Web of Science, and Google Scholar. The search covered publications from January 1981 to December 2024. The following keywords and Boolean operators were used in various combinations: “diquat poisoning,” “acute herbicide toxicity,” “Acute Diquat Poisoning,” “optimization of concentration-directed,” “supportive care AND herbicide,” and “renal replacement therapy AND toxicology.”

Inclusion criteria encompassed original research articles, case reports, case series, systematic reviews, and relevant clinical guidelines published in English. Articles were selected based on their relevance to the diagnosis, treatment, and clinical outcomes of acute diquat poisoning. Initial screening was performed by reviewing titles and abstracts, followed by full-text evaluation of selected studies. Studies lacking clinical relevance or adequate methodological quality were excluded.

Ultimately, the findings from the literature review were synthesized to provide context for the current case series and to highlight existing challenges and therapeutic strategies in managing diquat poisoning.

### 2.2 Blood purification procedure

For patients with diquat poisoning, blood purification therapy was performed based on their clinical severity, toxin levels, and organ function. The modalities applied included HP, continuous renal replacement therapy (CRRT), or a combination of both. HP was conducted using a cartridge (HA280, JAFRON Biotechnology Co., Ltd., China). Each session lasted 4 h, with blood flow rates of 150 mL/min. Systemic anticoagulation was achieved with unfractionated heparin, given as an initial bolus of 62.5–125 IU/kg followed by continuous infusion at 1,250–2,500 IU/h.

Continuous renal replacement therapy was carried out in a continuous veno-venous hemofiltration (CVVH) mode by using the blood purification machine (Diapact CRRT, Braun, Germany) with a high-flux hemofilter. The blood flow rate was maintained at 100 mL/min, and the replacement fluid rate was 150 mL/h. Each CRRT session lasted 4 h per day, depending on the patient’s hemodynamic tolerance and plasma diquat concentration.

For combined therapy, HP was typically performed first to rapidly adsorb circulating toxins, followed immediately (within 1–2 h) by CRRT to maintain continuous clearance and hemodynamic stability. The average number of total sessions was five per patient, based on the individual toxin levels, renal recovery, and clinical response. This combined therapy of HP and CRRT was guided by real-time monitoring of plasma diquat concentrations and multidisciplinary consultation, such as nephrology, intensive care unit (ICU), and emergency departments.

### 2.3 Supportive care and adjunctive therapy

The supportive treatments were given based on the patient’s specific condition. For patients with hypoxemia or respiratory distress, oxygen supplementation via nasal cannula or mask was carried out. For patients with severe hypoxemia, respiratory muscle fatigue, or altered consciousness who underwent endotracheal intubation, invasive mechanical ventilation using lung-protective strategies (tidal volume 6–8 mL/kg, PEEP 5–10 cmH2O) was necessary. The renal supports are also important for patients with diquat poisoning. Approaches such as fluid therapy, loop diuretics (e.g., furosemide 20–40 mg intravenous bolus, repeated as needed), and strict input/output monitoring were used to optimize renal perfusion and prevent volume overload. All patients received a combination treatment of reduced glutathione and vitamin C (2–3 g/day IV) for 7–10 consecutive days to mitigate oxidative stress. In some cases, N-acetylcysteine (NAC, 150 mg/kg loading dose followed by 50 mg/kg every 8 h, IV) was administered for 3–5 days as adjunctive antioxidant therapy, particularly in patients with elevated hepatic transaminases. Additional supportive measures included gastric protection with proton pump inhibitors (omeprazole 40 mg/day IV), broad-spectrum antibiotics for suspected infections, enteral or parenteral nutrition, and psychological support.

### 2.4 Analytical quantification of plasma diquat concentration

The plasma diquat concentration was measured to timely monitor of the drug level of patients by using liquid chromatography-tandem mass spectrometry (LC-MS/MS). Chromatographic separation was achieved on an Atlantis™ Premier BEH Z-HILIC column (2.5 μm, 2.1 × 100 mm) maintained at 40 °C. The aqueous phase consisted of deionized water containing 0.5% formic acid and 50 mmol/L ammonium acetate, while the organic phase was acetonitrile with 0.5% formic acid. A gradient elution was employed at a flow rate of 0.3 mL/min, with an injection volume of 2 μL. The mass spectrometer was operated in electrospray ionization positive mode (ESI?) using the multiple reaction monitoring (MRM) scan mode. For diquat detection, ion pairs with m/z 183–157 and 183–130 were monitored, with collision energies of 24 and 40 eV, respectively.

## 3 Case presentation

All experiments involving humans were conducted in accordance with the Ethical Standards of the National Ethics Committee and the Declaration of Helsinki 1964. This study was approved by the Ethics Committee of West China Hospital of Sichuan University (No. 1456, 2023). Informed consent was obtained from all participants included in this study.

### 3.1 Case 1

A 21-year-old male presented to our emergency department on 8 August 2022, after intentionally ingesting 200 mL of diquat the previous day. He had undergone two sessions of blood perfusion (unspecified) at another hospital with poor therapeutic effect. On admission, his heart rate was 122 bpm, respiratory rate 20 breaths/min, blood pressure 147/97 mmHg, and peripheral oxygen saturation (SpO_2_) 96%. Laboratory tests revealed a diquat plasma concentration of 3125.47 ng/ml, white blood cell count of 27.72 × 10^9^/L, platelet count of 164 × 10^9^/L, alanine aminotransferase (ALT) 92 IU/L, aspartate aminotransferase (AST) 174 IU/L, glucose 12.33 mmol/L, urea 9.5 mmol/L, creatinine 323.00 μmol/L, serum cystatin C 3.65 mg/L, creatine kinase 285 IU/L, lactate dehydrogenase 864 IU/L, prothrombin time (PT) 14.3 s, activated partial thromboplastin time (APTT) 123.5 s, pH 7.33, and albumin 27 g/L. The chest computed tomography (CT) showed scattered ground-glass opacities and cord-like shadows in the lower lobes of both lungs, decreased liver density suggestive of fatty liver, gastrointestinal fluid and gas accumulation with scattered air-fluid levels, and dilated colon.

He was diagnosed with pesticide poisoning (diquat poisoning), pulmonary infection, multi-organ dysfunction (heart, lung, kidney), fatty liver, metabolic acidosis, hypoproteinemia, and gastrointestinal bleeding. Despite initial treatment with blood perfusion and anti-infection therapy, his condition deteriorated and he was transferred to the ICU and nephrology department for further management. CRRT and HP were performed, combined with antioxidant therapy, anti-infection, enema, and catharsis. During this period, multidisciplinary consultations including nutrition, psychology, and rehabilitation specialists were arranged, and he was discharged on 12 September after obvious improvement. During the follow-up of 3 months, the blood concentration of diquat was undetectable, and there were no abnormalities in the blood routine, liver function, kidney function, or coagulation function.

### 3.2 Case 2

A 19-year-old female presented to our emergency department on 24 March 2023, with a history of intentional ingestion of 120 ml of diquat 10 h before. Her heart rate was 89 bpm, respiratory rate 19 breaths/min, blood pressure 131/85 mmHg, and SpO_2_ 92%. Laboratory tests showed a diquat plasma concentration of 5591.6 ng/ml, APTT 37.0 s, thrombin time (TT) > 120 s, glucose 6.84 mmol/L, creatinine 104.00 μmol/L, serum cystatin C 1.55 mg/L, hemoglobin 155 g/L, and white blood cell count 18.43 × 10^9^/L. The chest CT revealed inflammatory nodules in the left lower lobe of the lung. She was diagnosed with diquat poisoning and treated with symptomatic treatment, including cefmetazole, lactulose, and fluid replacement. She was then transferred to the ICU for further therapy, including catharsis, anti-infection therapy, antioxidant treatment, blood purification (blood perfusion and CRRT), and hepatoprotective and gastric protective approaches based on multidisciplinary consultations. On March 29th, the patient requested to be discharged voluntarily, and her survival condition was good during follow-up. Follow-up for 3 months showed normal blood routine, liver function, kidney function, or coagulation function, and the concentration of diquat was undetectable.

### 3.3 Case 3

A 23-year-old female presented to our hospital on 30 November 2021, with a history of ingesting 30–40 ml of diquat 2 days prior and vomiting a small amount afterward. Her heart rate was 89 bpm, respiratory rate 19 breaths/min, blood pressure 131/85 mmHg, and SpO_2_ 92%. Physical examination revealed pale skin and sclera, oral mucosal erosion, and non-pitting edema in both lower limbs. Laboratory tests on admission showed a diquat plasma concentration of 2475.1 ng/ml, hemoglobin 72 g/L, platelet count 328 × 10∧9/L, white blood cell count 18.26 × 10∧9/L, ALT 150 IU/L, AST 114 IU/L, urea 15.6 mmol/L, creatinine 654.00 μmol/L, glomerular filtration rate (GFR) 7.13 ml/min/1.73 m^2^, creatine kinase 14869 IU/L, and serum potassium 6.15 mmol/L. The chest CT scan showed a few cord-like shadows in both lungs and focal fatty infiltration in the left medial lobe of the liver. She was diagnosed with pesticide poisoning, multi-organ damage, rhabdomyolysis, acute renal failure, liver dysfunction, moderate anemia, and hyperkalemia. Treatments including diuretics, catharsis, fluid replacement, anti-infection therapy, CRRT combined with blood perfusion, and hepatoprotective measures were performed.

On December 2, her condition worsened and she was transferred to the ICU. Brain CT indicated brainstem infarction. Her respiratory rhythm became irregular and the SpO_2_ was decreased. Treatments, including reducing intracranial pressure, blood perfusion, antioxidant therapy, hepatoprotection, electrolyte regulation, endotracheal intubation, and CRRT combined with blood perfusion were given to her, and the multidisciplinary consultations, including nephrology, hematology, neurology, and other specialists were also arranged. Despite these efforts, she remained in a coma and was discharged on December 9 due to financial difficulties. The patient died on December 9th.

### 3.4 Case 4

A 14-year-old female with a history of depression for 1 year presented to our emergency department on 5 February 2022, approximately 14 h after intentional ingestion of diquat. The patient immediately underwent gastric lavage at another hospital. She experienced mild dyspnea and a burning sensation in her throat and upper abdomen. After admission, her heart rate was 70 bpm, respiratory rate 19 breaths/min, blood pressure 136/88 mmHg, and SpO_2_ 100%. Laboratory tests showed a diquat plasma concentration of 4512.26 ng/ml, ALT 112 IU/L, AST 216 IU/L, glucose 7.55 mmol/L, creatinine 214.00 μmol/L, GFR 29.12 ml/min/1.73 m^2^, uric acid 552 μmol/L, PT 9.2 s, APTT 22.2 s, and white blood cell count 17.76 × 10^9^/L. She had oliguria.

She was diagnosed with pesticide poisoning (diquat), and liver and kidney dysfunction. Treatment approaches, including antioxidant therapy, anti-infection measures, fluid replacement, and blood perfusion combined with CRRT were given. After treatment, the patient’s laboratory results were as follows: hemoglobin 62 g/L, white blood cell count 13.79 × 10^9^/L; creatinine 427 μmol/L, estimated glomerular filtration rate (eGFR) 12.63 ml/min/1.73 m^2^, myoglobin 66.41 ng/ml, NT-proBNP > 35,000 ng/L, and troponin T 30.2 ng/L. The plasma diquat concentration was undetectable. The patient had a low urine output and generalized edema. The patient was transferred to the Department of Nephrology with a diagnosis of “stage 3 acute kidney injury, pesticide poisoning, liver insufficiency, cardiac insufficiency, depression, gastrointestinal bleeding, and pulmonary infection.” After symptomatic supportive treatments such as anti-infection, diuresis, and blood purification, the creatinine level decreased significantly, and the patient’s condition stabilized. The patient was discharged on March 9th. At the 3-month follow-up after discharge, diquat was undetectable in the blood. Complete blood count, liver function, and coagulation parameters were within normal limits. Serum creatinine was 287 μmol/L with an eGFR of 20.63 mL/min/1.73 m^2^, and NT-proBNP was 900 ng/L, indicating chronic renal insufficiency and chronic cardiac dysfunction.

## 4 Discussion

Acute diquat poisoning is a serious and potentially life-threatening condition resulting from exposure to diquat, a widely used herbicide known for its rapid action in weed control. Acute diquat poisoning commonly occurs through ingestion via the digestive tract, where it is absorbed slowly in the intestines. Most of the ingested diquat is excreted through feces within 24 h, while a portion that is absorbed into the bloodstream primarily accumulates in the liver and kidneys. Since the kidneys are the main excretory organs, renal damage is particularly prominent. Acute kidney injury is one of the hallmarks of acute diquat poisoning, which can manifest as proteinuria, hematuria, or pyuria, and may progress to renal failure in the later stages.

The early symptoms of acute diquat poisoning are similar to paraquat poisoning, mainly characterized by corrosive effects on the oral mucosa, esophagus, throat, and abdominal cavity. The brain and spinal cord have physiological barriers that, to some extent, protect against the toxic effects of diquat. However, when the concentration of diquat accumulates to a certain level, it can cause central nervous system damage and reproductive developmental toxicity (4). Central nervous system poisoning symptoms are relatively common in acute diquat poisoning and may present as irritability, restlessness, delayed response, delirium, language disorders, or transient loss of consciousness (5). In addition to these symptoms, myocardial injury, rhabdomyolysis, and intestinal paralysis may also occur. In humans, the lethal dose of 20% diquat is 6–12 g. When more than 12 g of diquat is absorbed through the digestive tract, it can rapidly progress to multi-organ failure, leading to fulminant poisoning with a poor prognosis.

Currently, there is no specific antidote for acute diquat poisoning. Given the clear dose-effect relationship between the amount of diquat ingested and the prognosis of patients with diquat poisoning, rapid and comprehensive removal of toxins from the body is the key procedure in the treatment of acute diquat poisoning. According to the Expert Consensus on Diagnosis and Treatment of Acute Diquat Poisoning in China, blood purification is the primary measure to eliminate diquat from the bloodstream (6). The kidneys are the most common target organs for diquat, renal damage caused by diquat poisoning often leads to renal failure, and renal replacement therapy is an effective strategy. Besides, hemodialysis, HP, and CRRT are commonly used renal replacement therapies in clinical practice. Hemodialysis uses a dialysis device to allow toxic substances in the blood to diffuse into the dialysate, thus eliminating the toxins. HP involves the physical adsorption of toxic substances to compete with albumin binding, thereby removing toxic substances from the blood. HP is more recommended for patients with herbicide poisoning (7, 8). Ozaki et al. (9) reported that HP can improve clinical symptoms in patients, whereas the clearance rate of HP is limited; it cannot fully correct electrolyte and acid-base imbalances and may cause abnormal blood profiles. Moreover, the re-entry of toxins from the intestines, muscles, and fat into the bloodstream can lead to rebound effects (10). CRRT, also known as continuous blood purification (CBP), is a sustained blood purification method and one of the most used blood purification techniques in severe cases. It can continuously, stably, and slowly remove toxic substances, pathogenic factors, and inflammatory mediators from the systemic circulation, maintaining the stability of the internal environment for an extended period and reducing the occurrence rate of disease rebound and recurrence. However, CRRT has limitations, including contraindications, longer duration, and poor patient tolerance (11). It is reported that CRRT restores renal function faster than hemodialysis and provides hemodynamic stability, with no significant difference in mortality (12). In this case series, four patients with acute diquat poisoning were treated with a combined HP and CRRT regimen. Among them, three patients had favorable outcomes and survived; another one patient, who presented 14 h after ingestion, showed a gradual decrease in blood diquat concentration following the combined treatment. However, due to the delayed presentation, this patient developed multi-organ damage and eventually died.

Multidisciplinary collaboration is an effective strategy in clinical practice that optimizes the allocation of hospital resources and facilitates the timely treatment of patients, this novel model arranges the different specialties at various stages of the disease process. During the treatment of our patients, a multidisciplinary collaboration among emergency medicine, intensive care, nephrology, vascular medicine, surgery, rehabilitation, otolaryngology, and psychology was carried out, and held the principle of “treating the symptoms urgently and addressing the root cause gradually.” In the early stages, the focus was on rapidly removing unabsorbed toxins from the body and accelerating the excretion of absorbed toxins. In the later stages, the emphasis shifted to preventing multi-organ dysfunction and minimizing the toxic effects of diquat. Specifically, CRRT was initially administered to eliminate accumulated toxins, pathogenic factors, and inflammatory mediators, thereby stabilizing the internal microenvironment and protecting vital organ functions. This was complemented by enemas, catharsis, acid suppression and gastric protection, antioxidant therapy, and anti-infection measures. Throughout the treatment, close monitoring of the patient’s blood profile, biochemical parameters, organ function, and life quality was maintained. Comprehensive guidance on nutrition, rehabilitation, and psychology was provided, and the treatment plan was adjusted in real time based on changes in the patient’s condition. Collectively, multidisciplinary collaborative treatment for acute diquat poisoning not only optimizes the allocation of hospital resources but also maximizes the strengths of each specialty. This approach ensures that patients receive high-quality and comprehensive medical care, improving outcomes and overall well-being.

## 5 Clinical perspectives

Background: Acute diquat poisoning lacks a specific antidote and frequently leads to multi-organ dysfunction syndrome (MODS) with high mortality. Current blood purification therapies (e.g., HP, CRRT) lack individualized optimization, necessitating strategies to enhance toxin removal and improve outcomes.

Summary of results: In a series of four acute diquat poisoning patients, concentration-directed blood purification (combining hemodialysis, HP, and CRRT) guided by real-time plasma diquat monitoring significantly reduced toxin levels, improved organ recovery, and achieved survival in three cases. Multidisciplinary collaboration further optimized critical care support.

Significance: This approach demonstrates that dynamic, concentration-guided blood purification can enhance survival in acute diquat poisoning. It establishes a framework for personalized detoxification, potentially reducing MODS-related mortality and informing management protocols for other toxin-mediated critical illnesses.

## Data Availability

The original contributions presented in this study are included in this article/supplementary material, further inquiries can be directed to the corresponding author.
